# Observations on the Pathology, Symptoms and Treatment of Diseases of the Duodenum

**Published:** 1847-06

**Authors:** Samuel Salisbury

**Affiliations:** Avon Springs. Avon, Livingston Co., N. Y.


### BUFFALO MEDICAL JOURNAL

AND

### MONTHLY REVIEW.

VOL. 3. JUNE, 1847. NO. 1.

### ORIGINAL COMMUNICATIONS.

ART. 1.—*Observations on the pathology, symptoms and treatment of diseases of the Duodenum.* By SAMUEL SALISBURY, Jr., M. D., of Avon Springs.

*No.* 1. *Pathology.*—The frequent opportunities of investigating, diseases of the digestive organs which have been afforded me, among the numerous visitors of the Sulphurous Springs in this neighborhood, have led me to regard with particular interest, the disorders of this portion of the alimentary canal. From a minute inquiry into the early history and pathognomic symytoms of many different cases, I have formed the conclusion, perhaps erroneous, that one-third of those viewed and treated by their physicians as diseases of the Liver, have been in reality either functional or organic diseases of the Duodenum. Broussais long since advanced the opinion, in which Andral has rather concurred, that in most cases of inflamation of the Liver, there has been previous Duodenitis. Very recently Dr. Corrigan of Dublin has called the attention of physicians to the symptoms of the first stage of what has of late years been termed Cirrhosis of the Liver, a disease which consists in a chronic inflammation of the cellular tissue that forms Glisson's capsule; these he considers "as nothing more than the signs of Duodenitis. It is manifest however, that both Broussais and Andral looked upon this intestine as less liable to inflammation than other parts of the intestinal tube ; it is therefore to be presumed that they intend to include both functional and organic affections of the Duodenum under the appellation Duodenitis. The "cholic fits," or paroxysms of pain in the right epigastrium, which Dr. Carrigan describes, are symptomatic of functional as well as structural disease, and may proceed from irritation of the organic nerves by the passage of the ingesta. It is worthy of remark that he infers the gastric origin of Cirrhosis of the Liver from the fact that he found this disease curable by the same remedial measures which are successful in removing "certain functional diseases of the digestive organs." There is then reason to infer, from the observations of these distinguished pathologists ; not that inflammation or structural lesion of the Duodenum is a frequent occurrence ; but that irritation of its organic nerves, and an altered condition of the circulation, unaccompanied by inflammation ; in fact, what is called by Dr. Wilson Philip, *bilious dyspepsia*, and by Dr. Todd, *duodenal dyspepsia*, in a very large proportion of cases precedes organic disease of the Liver.

If it be admitted that the organic, or, as they have been sometimes called, the ganglionic nerves, possess properties which distinguish them from those of the brain and spinal cord, and this has been proved by recent experiments in regard to some of them, it is equally clear that the distinctive property of the former, is irritability. The first action of operative agents in the production of disease, such as heat, cold, infectious or poisonous matter, indigestable food, &c., must be, either upon the capillary vessels or nerves of the surface of the body or upon those of the mucous lining of those cavities which open externally, such as the lungs, alimentary canal, &c. The first effects of these agents must be an increase or diminution of the irritability of the nerves, or contractility of the vessels, of the parts on which they act. The Duodenum is supplied with branches of the same nerves as the stomach, liver, gall-bladder, and pancreas. The same mucous membrane is continued from the stomach to the Duodenum ; thence through the ductus communis to the liver and gall-bladder, and from the Duodenum to the pancreas. From these considerations it cannot be very difficult to conceive, that an increase of the irritability of the nerves, or of the contractility of the vessels, of this viscus may occur again and again, from the frequent reception of irritive ingesta, or other causes, and be propagated to its congeners, inducing irritation, inflammation, and even structural changes, in them, and yet no inflammation, or organic lesion, of this intestine take place. No matter whether this effect be thought to be produced immediately upon the nerves or vessels of the Duodenum, or by imbibition into the circulating current, it is in either sense irritation, and is not necessarily succeeded by inflammation. "Ribes suggested," says Dr. William Thompson,* (and Andral seems to have agreed in the opinion,) that inflammation commencing in the intestinal canal may propagate itself to the liver, not only along the mucous membrane, but also along the veins. It seems not impossible that inflammation of the Duodenum, without extending beyond the orifice of the ductus communis, may obstruct the flow of bile, so as to occasion in the first place jaundice, and eventually organic disease of the liver." Without calling in question, but rather coinciding with this opinion, there is reason for believing that the same effects may be produced upon the liver by functional disease of the Duodenum or Duodenal irritation. It is not necessary to imagine a patch of inflammation in the Duodenum, in every case, as the records of *post-mortem* examinations have clearly shown no portion of the alimentary canal being found, so frequently in sound condition. Mr. John Bell observes in his work on Anatomy, that it has been the opinion of the most respectable old physicians, those whose knowledge of diseases has been drawn from an acquaintance with anatomy, from the frequent inspection of dead bodies, and the observation of the symptoms during life, that the study of diseases connected with the Duodenum, is the most important which can occupy the attention of the medical inquirer.† It is certainly important to look to the earliest rational and physical signs of disorder in this viscus, as a knowledge of these may be considered a key to the diseases of the viscera with which it is immediately connected.

The anatomical position of the Duodenum, the part which it subserves in the process of digestion, and its organization, support

* Tweedie's Library of Practical Medicine, Diseases of the biliary organs, vol4.p242.

† Bell's Anatomy, vol. 4. p. 70., 1804.

the opinion that it is very liable to functional diseases or irritation, but that inflammation, or alterations of structure, are of rare occurrence. It is proper to explain here that when we speak of irritation we mean a mere alteration of the action of the nerves or bloodvessels of a part, without change of tissue. Tickle the throat with a feather, and irritation of the part, and of the stomach, is produced ; but were we to call this inflammation, then every abnormal action is inflammation, and the generally acknowledged distinction between functional and organic disease cannot exist.

The Duodenum occupies a position among the viscera which would seem to render it peculiarly liable to disease. It is a central organ which is most intimately connected with the stomach, liver, gall-bladder and pancreas. In its course it describes nearly a whole circle. Beginning at the pyloric orifice of the stomach, it passes backwards, and to the right side, touching the gall-bladder. It there crosses and re-crosses the spine, enclosing one end of the pancreas in the concavity of its curve. It is the receptacle of the chyme from the stomach and the secretion of the liver and pancreas. In this intestine principally it is thought such a change must be effected in the chyme, sometimes imperfectly formed, as to convert it into the mild and unirritating substance which we denominate chyle. Irritate the extremity of the common duct which opens into the Duodenum and it is felt by the stomach, liver and pancreas through their whole extent, and the proper elaboration of chyle is prevented. As a secondary effect of such irritation deficient nutrition must necessarily result. In order to prevent such exigencies we find in this intestine a peculiar organization, giving it, as we believe an extraordinary power resisting and throwing off the impressions of deleterious agents.

I. It is to be observed that it is susceptable of great distension. *Post-mortem* dissections have revealed to us the fact that it is sometimes distended and enlarged to such a degree as to equal the stomach in size. This can only be explained by supposing its several coats possessed of great strength and elasticity.

II. The mucous coat is thicker than that of any other portion of the alimentary tube, in the upper two-thirds of this intestine. The villi with which this coat is covered are very numerous.

These villi, according to the recent observations of Mr. Goodsir, become turgid and erect every time that chyme passes into the intestine, and the epithelium covering them, is cast off, so that the chyme can come in immediate contact with their exposed surface. They are furnished with numerous cells, formed from the epithelia lining them, which, when they have drawn into their interior certain suitable materials from the chyme, burst and discharge their contents into one or two lacteals with which the villi are immediately connected. When this absorption is not going on, the epithelia covering the villi protect their surface from the acrimony of the fluids constantly coming into the intestine from the ductus communis.*

III. The sub-mucous cellular tissue, which was called by ancient anatomists, the nervous coat, is worthy of special attention. Embedded in its substance are the mucous follicles or glands of Brunner, which are more numerous and much larger in the Duodenum than in any other portion of the intestinal tube, being twelve times larger than those of the stomach, and so prominent as to give the lining membrane a granulated appearance. The office of these glands is supposed to be, the secretion of a fluid to lubricate the inner surface of the intestine and perhaps the elaboration of a distinct product. Both in acute and chronic Duodenitis, these glands are found to be turgescent and hypertrophied.

IV. The muscular coat too, is thick, the circular fibres being particularly strong. Dr. Macintosh in his Principles of Pathology expresses a doubt whether acute inflammation ever primarily effects this coat of the bowels ; but rather extends itself to it from the mucous or serous coats. In inflammations of the mucous coat he observes, there is generally diarrhœa ; but when the muscular tunic is in a state of inflammation, there is obstinate constipation. To apply this remark to disease of the Duodenum, the occurrence of diarrhœa is very unfrequent, and it is almost always attended with a greater or less degree of constipation. A reasonable inference from this fact is, that the muscular tissue is the usual seat of chronic inflammatory action, the mucous and sub-mucous tissues being protected by their extraordinary thickness, and the great number and size of their villi and glands.

*Anatomical and Pathological Observations, by John & H. D. Goodsir. 1845.

"The relative frequency of *acute* inflammation is much greater," says Dr. Gross in his Pathological Anatomy, "in some parts of the alimentary canal than in others. The lower half of the ileum, the stomach, and the commencement of the colon, are without doubt most commonly engaged, either alone, or conjointly." It has been suggested by the same writer that the Congestive Fever of the Southern States may be caused by acute inflammation of Duodenum, being propagated from this intestine to the liver and gall-bladder; but post-mortem inspection which often reveals traces of inflammation in collatitious viscera sustains the position above taken, by showing that the stomach and liver may be both together in a state of inflammation and yet the Duodenum experience merely irritation or functional disease. Very recently an epidemic disease is said to have prevailed in some parts of the Northern States (see the March number of this Journal,) the symptoms of which are those indicative of disease of the Duodenum. It is unfortunate that there have been no post-mortem examinations to ascertain whether the disease was functional or organic, so far as this viscus was implicated. We are not informed whether in any instance the disease proceeded to a fatal termination.

Avon, Livingston Co., N. Y.

